# A Qualitative Study of an Employment Scheme for Mentors with Lived Experience of Offending Within a Multi-Agency Mental Health Project for Excluded Young People

**DOI:** 10.1007/s11414-018-9615-x

**Published:** 2018-05-31

**Authors:** Eleanor Hodgson, Jenny Ruth Stuart, Charlotte Train, Michael Foster, Leon Lloyd

**Affiliations:** 1 MAC-UK, London, UK; 2 NHS, London, UK

## Abstract

The use of peer intervention schemes is increasingly popular within services for offending groups and there is growing evidence of their benefit for peers delivering the interventions. The current study explores staff and peer experiences of an employment scheme for peer mentors within a community project for young people involved in offending. Semi-structured interviews were conducted with full time staff (*n*=4) and peer mentors employed on the scheme (*n*=2). Thematic analysis revealed three themes of “Opportunity and Empowerment”, “Supportive Processes” and “Role Definition and Structure”. The results suggest that meaningful employment opportunities, with holistic support and processes for reflection and mentalisation of self and others, contribute to the personal and professional development of ex-offenders in peer mentoring roles.

**Electronic supplementary material:**

The online version of this article (10.1007/s11414-018-9615-x) contains supplementary material, which is available to authorized users.

## Introduction

The use of ‘peer intervention’ schemes is increasingly commonplace in a wide range of settings including schools, prisons and mental health services.^1^^,^^2^ The philosophical development of peer intervention programmes is rooted in the community psychology principles of social justice and empowerment.^3^^,^^4^ Peer intervention is perceived as a means to utilise, respect and value the lived experience of often marginalised individuals for the mutual benefit of those involved as well as the wider community. Literature on the efficacy of peer intervention schemes for both the recipients and the peers themselves is still emerging, with much of the evidence published in in-house reports.^5^^,^^6^

Benefits of peer intervention programmes for those in peer roles include enhanced career prospects and increased employability skills, increases in measures of empowerment such as self-efficacy, sense of power and optimism about the future, and personal development in the form of increased confidence, understanding of self, managing emotions and relationships with others.^2^^,^^7^^,^^8^

Research has recognised a range of challenges and complexities in peer intervention programmes, as well as the benefits. Fletcher and Batty note that the level of experience and skill to deliver a peer intervention is high; requiring good communication skills, the ability to empathise and assume a non-judgemental stance.^9^ As such the authors highlight that mentors are likely to require substantial support. Mowbray et al. found that taking on a ‘peer-supporter’ role could entail a cost to the peer’s own wellbeing through increased stress and difficulty in knowing what to do in the role.^2^ A number of studies found that mentors have struggled with managing boundaries and confidentiality with clients.^2^^,^^6^^,^^10^^–^12 Peer relationships with clients may be complex for a number for reasons including pre-existing personal relationships with clients, and service expectations of disclosure of ‘service-user’ status.^10^^,^^11^ This situation can place peers in a grey area in which they are both simultaneously and neither ‘service users’ and professionals.

These personal and professional challenges exist within organisational contexts which often lack adequate training, support and supervision as well as necessary policies to support the definition of mentor roles.^11^^–^15 Another organisational challenge has been identified as attitudes of non-mentor staff, including negative attitudes towards recovery and the ability of mentors to perform in the role.^13^ Wolf and colleagues outline three levels of infrastructure emerging as best practice from the mental health field: provision of pre-employment preparation and training; well-defined human resources policies and procedures; and a commitment to peer provision by leadership that is reflected in operations throughout an organisation.^16^

UK government policies have promoted the use of peer intervention for offenders.^17^^,^^18^ Yet, research of best practice for employing ex-offenders as peer mentors is limited. One avenue towards this would be to explore whether Wolf and colleagues’ best practice guidelines apply to peer mentoring schemes for ex-offenders.^16^ Qualitative research into the experiences of staff and peer mentors involved in such a scheme could contribute to the developing understanding of what is effective for peer mentors with lived experience of offending, and the systems around them, to overcome identified challenges.

Thus, the current study focuses on an employment scheme for peer mentors embedded within a multi-agency pilot of the ‘Integrate’ model. The pilot was led by the charity MAC-UK in partnership with a NHS trust and the local authority. The Integrate model is an approach to mental health service development and design for excluded young people and is described in detail elsewhere.^19^ The model aims to reduce offending, improve young people’s mental health, support access to services and employment, and create change at wider levels. The target group for this particular project were people aged 16–25 who were involved in serious offending behaviours. Attendees of the project were referred to as ‘young people’ within the project and throughout this article.

Core features of the Integrate approach include the application of Adolescent Mentalization-Based Integrative Therapy (AMBIT) and of community psychology theory and practice.^4^^,^^20^ AMBIT proposes that mentalisation (the process of trying to understand the thoughts, feelings and intentions of self and others) and building reflective capacity is central to effectively working with ‘hard to reach’ young people and their networks. AMBIT techniques are therefore built into the processes of staff teamwork, supervision and the clinical approach within the project.^20^^,^^21^

In line with community psychology theory and practice the project was co-produced with the local community. Part of this co-production model was achieved through an employment scheme with two tiers of opportunities. The first tier consisted of one-off or short-term employment which could be offered to young people in a variety of roles supporting the running of activities at the project (e.g. organising an event; decorating the project base). The second tier, and the focus of this paper, was the provision of longer-term part-time or sessional employment as a ‘peer mentor.’ This was offered to a small number of older members of the community (25–30) with lived experience of exclusion and offending, who were well respected and influential within the group of young people the project wished to engage. Each peer mentor role was tailored to the mentors’ strengths and interests but all roles included engaging young people and helping to co-produce and run activities at the project. Employment as a peer mentor required completion of mandatory training and attendance at regular supervision.

Community psychology theory also informed the approach to the current research and the research question was defined through collaborative discussion with all participants: ‘Which features of our employment scheme have contributed to the personal and professional development of peer mentors working in an integrated mental health and youth work service?’ It is acknowledged that the question contains an assumption that the scheme had a positive impact; however, it was considered appropriate as it reflected the experience and participation of the mentors currently on the scheme and who acted as co-researchers.

## Method

### Design

A participatory research approach was taken in which all participants were involved in the development of the overall research design.^22^ A qualitative design of individual semi-structured interviews was analysed using thematic analysis.^23^ An intensity sample of those with extensive experience of the peer mentor employment scheme was taken in order to obtain rich data for analysis. The interview schedule was designed, conducted and analysed by a trainee psychologist on placement at the project who received external research supervision. The interview schedule consisted of seven questions that allowed exploration of personal and professional development (for example: Has the employment scheme impacted the employees personally? Possible follow up/prompts: Wellbeing; Opinions/thoughts; Feelings/emotional states; Behaviours/activities; Relationship with self; Relationship with others).

### Participants

All permanent full time staff members (*n*=4; one mental health nurse, one clinical psychologist, and two youth workers) and two regularly employed part-time peer mentors (*n*=2) were approached to take part and all gave consent. At the time of interview all participants had been in their roles for a minimum of 2 years.

### Procedure

The information sheet was distributed to participants 3 weeks prior to scheduling of interviews. At the beginning of each interview any questions were answered and formal consent sought and recorded in writing. Interviews were audio recorded using a digital voice recorder and lasted between 35 and 60 min. Interviews were conducted in a private room at the project base.

### Data Analysis and Respondent Validation

Thematic analysis was used following the six phase process outlined by Braun and Clarke.^23^ An inductive approach was applied to allow the development of themes with strong links to the data identified at a semantic level.^24^^,^^25^ Analytic memos and thematic maps were used to support the process.^23^ Familiarisation with the data (phase 1) was achieved through transcription and repeated re-reading. In generating initial codes (phase 2), the first interview transcript was coded line-by-line and the remaining five were coded using larger text excerpts (sentences or short paragraphs) resulting in hundreds of codes which were then collapsed into 73 focused codes. Focused coding from two transcripts were checked with the corresponding participants; minor amendments were made in conjunction with reference to the original data. In searching for themes (phase 3) focused codes were collated and sorted into conceptually similar groups which produced 15 initial sub-themes. A similar process then followed in reviewing themes (phase 4) where the sub-themes were sorted until they formed four groups which were named and considered as overarching themes.

At this stage, the analysis was shared with participants and feedback supported phase 5 of defining and naming themes. In a process of referring back to the original codes and data and noticing similar content some of the 15 sub-themes were joined together to form 11 sub-themes. This process was also used to collapse the original four overarching themes into three overarching themes. This resulted in overarching themes that were conceptually different from one another, and internally were made up of related codes and sub-themes. The description of these themes in the results and discussion below completed the analysis (phase 6; producing the report).

### Quality assurance

In addition to respondent validation as described above, Braun and Clarke’s 15 point checklist of criteria for a good thematic analysis was used in effort to ensure quality (Table 1, electronic supplementary material).^23^ External research supervision was used to provide independent checks on coding as well as supporting the analysis through discussion of the process and developing themes.

## Results

Three overarching themes were identified from the data: ‘Opportunities and Empowerment’, ‘Supportive Processes’ and ‘Role Structure and Definition.’ Each theme contains sub-themes and is described below using anonymised direct quotations.

### Opportunities and empowerment

All of the participants talked about ways in which the employment scheme provided opportunities and empowered mentors. These appeared to take the form of an Alternative to Offending, an Opportunity to be Valued, and the Opportunity to ‘Bridge Out’ from the scheme to other employment opportunities.

#### Alternative to offending

Participants seemed to promote an understanding of offending in terms of lack of alternative options and opportunity for making money. The employment scheme was commonly described as for ‘slightly older peers from the local community, who have been involved in offending, but have stopped, or want to stop, and might be looking for work but struggling to get it’ (participant (p) 3) and providing ‘someone employment, a sense of achievement and purpose in a less risky way, than may have been happening before’ (p1).

Participants spoke about the importance of experiencing an alternative (rather than knowing in principal or being told) in order to ‘understand what their options are’ (p4). A common theme was the importance of the opportunity being for ‘meaningful employment’ (p3) related to the person’s skills and interests, rather than employment per se.

#### Opportunity to be valued

Participants described how ‘the project wouldn’t exist without them [mentors], they are a very necessary part of the process’ (p2) and how having mentors working alongside permanent staff members ‘is why they [young people] come…they value those relationships, and it’s why the model was set up in the way it is’ (p6). Participants spoke about the opportunity to be valued acting as a bridge between young people and staff:

We [mentors] often know more about what is going on, what they [young people] are not really saying out loud-you know a lot of people talk in their music and I was able to pick up a lot of signals, and pass that onto my team (p5).

Participants also spoke about being valued for additional skills over and above being a link between young people and staff:

It’s like learning to value yourself for more than what you were valued as originally… unfortunately within the real world we don’t really tend to value ourselves that much-so, to be put in a place where you can be in the real world and valued, it’s a big eye opener (p4).

#### Bridging out

The final sub-theme of the overarching Opportunity and Empowerment theme was given the name ‘Bridging Out’. This term is used within the Integrate model to describe a process of supporting connections with external opportunities and agencies related to employment, health and social needs. Being involved in the employment scheme appeared to be an opportunity to support this process through increased skills, knowledge and confidence:

I think its strengthened them big time, from where they were at the beginning to where they are now, its opened up the realms of possibility about what can be done and what can be achieved, whereas someone may have just thought this is the only thing they can do, to maybe I can do this-I can do that, (p1).

Other participants had ideas about how the process of bridging out might be different and one participant mentioned that the scheme could be shorter to avoid building dependency on the project:

It’s hard to know within this model, because everything is youth led…but, I wonder if it could be an idea to have three months of this, three months of that … and by the end of the six months you’d be looking for full time employment or whatever it is, so you don’t get too comfortable or dependent (p6).

### Supportive processes

The second overarching theme was called ‘Supportive Processes’ as it captured a range of informal and formalised structures that supported the employees on the scheme, both personally and professionally. These included ‘Training and Learning’, ‘Supportive Professional Relationships and Environment’, ‘Personal Support’ and ‘Reflective Supervision’.

Some participants spoke about ‘the amount of support that’s needed to really enable people to function in those roles is really big, and I think that’s right. It’s important and we’ve prioritised that because we think it’s important, but I guess that doesn’t mean that it’s a cheap option, and I suppose I wouldn’t want it to be a really good thing if it was’ (p2).

#### Training and learning

All of the participants spoke about the training and learning opportunities available to those on the employment scheme: ‘we’ve put some money and flexibility in to enable them to do CPD type opportunities like, take a course, learn, and attend training’ (p2).

Participants described the perceived benefits of training for the peer mentors and the difference in their professional skills as a result:

Some of the conversations that were had, and are now are being had are different, they’re more attuned-they are having more therapeutic conversations like, thinking about sleep in terms of going to some of the IAPT workshops-bringing the information back, relaying that to young people. At the beginning it was all about studio, how do you be a rapper, how do you find out how to do that, whereas now it’s more about the process (p6)

One participant reflected on their own personal learning from professional training:

You learn about yourself, being in these training opportunities… I find that the training schemes don’t necessarily give you much new information; they give you ways to acknowledge what you do already, and then the words to explain it (p4).

#### Supportive professional relationships and environment

All the participants spoke of the utility of more informal support structures: ‘it’s useful to just check in as and when needed’ (p2). Participants also discussed the impact of working in a professional environment:

You may be able to relate to someone, or be in a relationship with someone on an informal basis but, working in a professional environment you get to meet different people from different walks of life and that may help someone to, yeah, support different needs, be able to meet new people, um all these things are of benefit in being able to work with others (p1).

I think just being within a team that thinks about wellbeing and is kind of considerate to others and is always trying to share and be explicit and support each other, I think is just helpful (p6).

#### Personal support

In addition to professional opportunities all peer employees are offered personal support which ranged from practical issues to more therapeutic support.

A lot of things in their lives-sort of instability, I don’t know not having a house, not having enough money to get to work, are kind of personal issues but without addressing them they’re not going to be able to successfully complete their employment so we’ve put mechanisms in place to support them with those things (p3)

Participants spoke about the impact of therapeutic support in developing an understanding of self and others, and the benefit on peer mentors’ personal lives and relationships as well as their work with young people and the team.

I never used to bear people’s opinions and thoughts and feelings in mind, I would retaliate because I wanted to, it was my normal chain of thought, and now I believe that anybody could do something for any given reason, but if you don’t take the time to find out what the reason is, then you never actually get to the bottom of anything (p4).

#### Reflective supervision

Participants spoke about the provision of ‘staff meetings, supervisions, group supervisions, team meetings, and we have team briefing’ (p5) as spaces to reflect on and discuss aspects of the work. All of the participants spoke about the importance of supervision and suggested that this needed to be a reflective space:

Going from having experiences that are so close to the young people that you are working with, you need to have an opportunity to work that out and what that feels like because, it can be really dangerous like, in terms of being able to mentalize the young person, because you’re so aroused by what’s going on for that young person because it’s close to what you’ve experienced…but having a space like supervision to, kind of unpick all of that, is really key and its I think what will make a successful peer mentoring scheme (p6)

### Role structure and definition

This overarching theme described some of the systemic context and structure of the scheme and was made up of four sub-themes that related to ‘Clarity’, ‘Flexibility’, ‘Role Boundaries’ and ‘Wider Support’ for the employment scheme.

#### Clarity

One aspect of the scheme that was described as challenging was clarity about the scheme itself; the aims, intentions and expectations:

To be honest I didn’t know that I was in that [employment scheme] for a while and me and another party were quite confused about what they were trying to accomplish in the start… I was never aware of actually being on a scheme (p5).

The boundaries are so blurred in the first place…like the bar is continually being lifted, so it goes from you are practically a young person with an opportunity to do some volunteering to now ok you’re employed, and these are the things we expect (p6).

Participants seemed to suggest that had these things been clearer from the beginning this would have supported the process of being employed as a mentor:

From the beginning it wasn’t necessarily well set out in terms of what the employment would look like, whether the expectations would shift, whether they would stay the same. I think we’ve learnt a lot from that-about needing to be clear from the beginning (p3).

#### Flexibility

Participants noted that in order for the scheme to be a viable opportunity it needed to be a ‘more flexible method of employment’ (p3) in which support processes were individualised ‘depending on what their needs are and what they want to address’ (p3). Participants spoke about the need to allow time in order for those on the employment scheme to develop trust within the team and understanding of the role.

One participant described how such an employment scheme is ‘going to have its challenges, people have their ways and sometimes being more flexible helps…you’ve just got to try and work around them [challenges]-or overcome the challenges or change, or manage’ (p1). Another participant also described the limits of flexibility, ‘it’s hard to manage if someone doesn’t actually … commit to the parts of the scheme that we think are really important, like supervision, how much can they dictate…and how much can’t they?’ (p2). One participant described how “not all workplaces will be as forgiving” (p6).

#### Boundaries

All of the participants described challenges in relation to various personal and professional boundaries. Participants described the difficulty in managing the boundary between being a part of the community versus being an employee, and the different perceptions people can have of a person in different roles, ‘they are part of the community and the young people see them in a different light, that maybe hard for some to manage, or balance’ (p1).

Participants spoke about the boundaries of the role of mentor as distinct from that of full time staff and what that meant for their roles with young people, confidentiality and information sharing, and their place within the team. Participants described a process of learning and negotiation that supported the definition of these boundaries:

Sharing information across teams, noticing our specialisms and why we are different and kind of learning together, so I think settings like group supervision kind of help (p6).

I spoke out about not attending the team meetings in the beginning, which actually resulted in us attending the team meeting (p5)

Full time staff also spoke about the boundaries around peer mentors also being young people who are in receipt of personal support:

It’s been a challenge from my perspective navigating that dual relationship, knowing when to be what and how to do that- how to be explicit about it…building the relationship to a point where its ok to kind of talk about that…was a lot of work in and of itself (p2)

#### Wider support

This final sub-theme included thoughts about the wider structural supports needed to make an effective employment scheme. Participants often spoke about the problematic nature of using zero-hour contracts:

Having zero hours contracts aren’t ideal but so we’ve offered them…set time, same days so then at least they can then plan around that (p3)

One participant thought that it would have been beneficial had their contract been more full time, ‘if we were given the opportunity to have full time jobs and commit more … the time it took to learn would have been cut in half’ (p5).

Some full time staff spoke about a lack of organisational policies or structure in place to support them in managing the challenges of the role:

We’ve had to kind of adjust things in relation to how people were performing but…it’s difficult to make those decisions without a structure in place to inform how you do that and it can-I guess it runs the risk of feeling kind of personal (p2)

Others suggested that if more wider system support were available this would create a more effective employment scheme, ‘on a government level if the government wants things to improve, then they need to show people that improvement is worth something’ (p4).

## Discussion

The current study set out to examine what features of the employment scheme contributed to the professional and personal development of peer mentors working in an integrated mental health and youth-work setting. The findings indicate 3 broad themes, each made up of a set up sub-themes: Opportunities and Empowerment which was comprised of Alternative to Offending, an Opportunity to be Valued, and the Opportunity to ‘Bridge Out’ from the scheme to other employment opportunities; Supportive Processes which was comprised of Training and Learning, Supportive Professional Relationships and Environment, Personal Support and Reflective Supervision; and Role Structure and Definition which was comprised of Clarity, Flexibility, Boundaries and Wider Support.

The Opportunity and Empowerment theme describes the quality of the experience of being on the scheme in contrast to previous experiences of deprivation and disempowerment. The importance of opportunities being genuine and meaningful is present across the sub-themes. Having a lived experience of an alternative to offending, in which employees felt valued and respected was perceived as a powerful experience which conferred a sense of value for self and hope for the future for the mentors.

This is consistent with Rogers et al.’s finding of the impact of peer intervention on measures of empowerment.^8^ This is also in line with conclusions and recommendations within the literature about ‘meaningful rather than tokenistic’ employment of peers and the importance of respect and commitment to peer intervention roles by full time staff. 9^,^^13^^,^^16^

The theme of Supportive Processes encompasses a range of structures which were identified as supportive of mentor’s development. Formal supportive structures included various meetings, supervisions and training opportunities. The Supportive Professional Relationships and Environment sub-theme reflected the learning that happened implicitly through the experience of being employed, working alongside other professionals and the supportive milieu of the project. Learning about and understanding self and others, both through explicit teaching and through the experience of relationships, is found across the Supportive Processes theme. This reflects the team’s use of AMBIT as a framework to increase mentalisation through all aspects of the project and the nature of the teamwork.

The theme highlights the inevitable interrelation between personal and professional development; for example, it was acknowledged that personal issues, both practical and emotional, may act as barriers to development within the role. In response to this interrelation participants emphasised the need for a holistic approach for the scheme to be successful. Previous research has identified the importance of supervision in peer intervention programmes in mental health settings.^11^^,^^14^^,^^15^ The supervision provided on the current scheme was influenced by the mental health specialism of the service and therefore was ‘clinical supervision’ provided by mental health professionals.^26^ The value placed on supervision by mentors, combined with the emphasis on supervision as a space to explore the connections between personal and professional life, extends the evidence base indicating this to be an important feature for peer intervention schemes in services working with mentors with a lived experience of offending.

The findings are in line with existing literature which emphasises the need for training and support to perform the role.^9^^,^^12^^,^^16^ It is worth noting that provision of adequate training, support and supervision are features one would expect to see for any roles within mental health or youth-work settings. Therefore, in principal this is no different from the structures that adequately support and develop any staff. However the findings highlight that the amount of support required may exceed that of other staff, and that there may be greater complexity of issues around supervision and management for peer staff. These findings therefore have implications for resources needed to adequately provide peer intervention programmes.

The final set of sub-themes from Role Structure and Definition highlight some of the tensions within the employment scheme. Clarity is described as something that was lacking when the scheme was first implemented, resulting in uncertainty for both full time staff and mentors about expectations of the role and the ultimate aims of the employment. This is also reflected in the sub-theme of Wider Support in which lack of structures or policies are cited as a challenge. Flexibility is raised as a requirement in order for the scheme to work and be responsive to the individual development needs of the mentors; but also a challenge in deciding on the limits of such flexibility. These tensions replicate previous research in the mental health field which have identified issues of role clarity and organisational/HR structures as common challenges of peer intervention programmes.^11^^,^^13^^,^^16^

Role Boundaries is a complex sub-theme in which relationships that the mentors have with both staff and young people contain challenges of definition and management of confidentiality. This is consistent with research which has identified a range of challenges in managing the boundaries for those in peer mentor roles.^2^^,^^6^^,^^10^^,^^12^ There is a question as to whether challenges in definition of boundaries is inherent to roles in which pre-existing relationships with individuals or communities are both utilised and adjusted through the intervention.

Throughout the Role Structure and Definition theme, the principle of being explicit in communication, through a process of active mentalisation, is seen as a way to manage these tensions. This therefore links back to the Supportive Processes which facilitated the mentors developing ability to think about, understand and navigate complex boundaries and relationships. This also reflects the use of AMBIT by the team and in supervision as a process of sharing thoughts and feelings and broadcasting intentions.

The current research has a number of limitations. The analysis was completed by a single researcher and therefore the study lacks inter-coder reliability. However, an iterative process of checking coding with participants and the use of external supervision were undertaken to provide quality assurance. The use of a researcher working at the project may have introduced a degree of reporting bias. However in participatory research the need for a safe space in eliciting rich and full responses is also emphasised.^22^ Therefore the benefit of having a known and trusted researcher may balance out this limitation. The interviews collected the views of two long-standing mentors. The sample therefore does not capture experiences of those who had shorter-term employment or left the scheme. Future research and evaluations of peer interventions should seek to include a wider range of employees and may benefit from more longitudinal or pre and post measurement designs.

## Implications for Behavioural Health

The current research contributes to the existing literature demonstrating the transformative potential of meaningful and genuinely valued employment for mentors with lived experience of offending. These findings support much of the previous literature on mental health peer intervention schemes and contribute to an emerging body of evidence on best practice to inform policy and service delivery.^16^ Clinical supervision and the AMBIT framework supported the delivery of holistic and trailered support, training and reflective practice; all of which were found as features which promoted personal and professional development. Processes of active and open mentalisation of self and others within the team were identified as helpful in managing the challenges of role definition and boundaries. This has implications for the framework used, and supervision provided, in peer intervention schemes for ex-offenders.

## Electronic Supplementary Material

ESM 1 (DOC 35 kb)

## References

[1] Devilly GJ, Sorbello L, Eccleston L (2005). Prison-Based Peer-Education Schemes. Aggression and Violent Behaviour..

[2] Mowbray CT, Moxley DP, Collins ME (1998). Consumers as Mental Health Providers: First-Person Accounts of Benefits and Limitations. The Journal of Behavioural Health Sciences and Research..

[3] Campbell, J. The historical and philosophical development of peer-run programmes. In Clay S, Schell B, Corrigan PW et al (Eds) *On Our Own, Together: Peer Programs for People with Mental Illness.* Nashville (TN): Vanderbilt University Press, 2015pp17-66.

[4] Kagan C, Burton M, Duckett P (2011). *Critical Community Psychology*.

[5] Hunter G, Kirby A (2011). *Evaluation of Working One to One with Young Offenders*.

[6] Pitts J (2006). *An Evaluation of the X-It Gang Desistence Programme*.

[7] Princes Trust and Institute for Criminal Policy Research. *Evaluation Summary: Working One to One with Young Offenders*. The Prince’s Trust, 2011.

[8] Rogers ES, Teague GB, Lichenstein C (2007). Effects of Participation in Consumer-Operated Service Programs on Both Personal and Organizationally Mediated Empowerment: Results of Multisite Study. Journal of Rehabilitation Research and Development..

[9] Fletcher DR, Batty E (2012). *Offender Peer Interventions: What Do We Know?*.

[10] Fisk D, Rowe M, Brooks R (2000). Integrating Consumer Staff Members into a Homeless Outreach Project: Critical Issues and Strategies. Psychiatric Rehabilitation Journal..

[11] Gates LB, Mandlberg JM, Akabas SH (2010). Building Capacity in Social Service Agencies to Employ Peer Providers. Psychiatric Rehabilitation Journal..

[12] Foster J (2011). *Peer Support in Prison Health Care. An Investigation into the Listening Scheme in One Adult Male Prison*.

[13] Gates LB, Akabas SH (2007). Developing Strategies to Integrate Peer Providers into the Staff of Mental Health Agencies. Administration and Policy in Mental Health Services Research..

[14] Walker S, Avis M (1999). Common Reasons Why Peer Education Fails. Journal of Adolescence..

[15] Hunter G, Kirby A (2011). *Evaluation of Working One to One with Young Offenders*.

[16] Wolf J, Lawrence LH, Ryan PM (2010). Emerging Practices in Employment of Persons in Recovery in the Mental Health Workforce. American Journal of Psychiatric Rehabilitation..

[17] Public Health England. *The Mental Health Needs of Gang-Affiliated Young People*. Public Health Institute, Liverpool John Moores University, 2015.

[18] Home Office. *Ending Gang Violence and Exploitation*. 2016. Available online at https://www.gov.uk/government/publications/ending-gang-violence-and-exploitation Accessed June 21, 2015

[19] Zlotowitz S, Barker C, Moloney O (2016). Service Users as the Key to Service Change? The Development of an Innovative Intervention for Excluded Young People. Child and Adolescent Mental Health..

[20] Bevington D, Fuggle P, Fonagy P (2012). Innovations in Practice: Adolescent Mentalization-Based Integrative Therapy (AMBIT) - A New Integrated Approach to Working with the Most Hard to Reach Adolescents with Severe Complex Mental Health Needs. Child and Adolescent Mental Health..

[21] Bevington D, Fuggle P, Fonagy P (2015). Applying Attachment Theory to Effective Practice with Hard-to-Reach Youth: the AMBIT Approach. Attachment & Human Development..

[22] Bergold J, Thomas S (2012). Participatory Research Methods: A Methodological Approach in Motion. Forum: Qualitative Social Research..

[23] Braun V, Clarke V (2006). Using Thematic Analysis in Psychology. Qualitative Research in Psychology..

[24] Boyatzis RE (1998). *Transforming Qualitative Information: Thematic Analysis and Code Development*.

[25] Patton MQ (1990). Qualitative Evaluation *and Research Methods*.

[26] Care Quality Commission. *Supporting Information and Guidance: Supporting Effective Clinical Supervision.* CQC, 2013.

