# Supplementary material for: A Qualitative Study of an Employment Scheme for Mentors with Lived Experience of Offending Within a Multi-Agency Mental Health Project for Excluded Young People
Source: J Behav Health Serv Res. 2018 May 31;46(1):140–50. doi: 10.1007/s11414-018-9615-x (PMC6323077; doi:10.1007/s11414-018-9615-x)
Supplement: Supplementary file 1 — (DOC 35 kb) [file 11414_2018_9615_MOESM1_ESM.doc]

Table 1. A 15-Point checklist of criteria for Good Thematic Analysis

| Process | No | Criteria |
| --- | --- | --- |
| Transcription | 1 | The data have been transcribed to an appropriate level of detail, and the transcripts have been checked against the tapes for ‘accuracy’ |
| Coding | 2 | Each data item has been given equal attention in the coding process |
|  | 3 | Themes have not been generated from a few vivid examples (an anecdotal approach), but instead the coding process has been thorough, inclusive and comprehensive |
|  | 4 | All relevant extracts for each theme have been collated |
|  | 5 | Themes have been checked against each other and back to the original data set |
|  | 6 | Themes are internally coherent, consistent and distinctive |
| Analysis | 7 | Data have been analysed – interpreted, made sense of – rather than just paraphrased or described |
|  | 8 | Analysis and data match each other – the extracts illustrate the analytic claims |
|  | 9 | Analysis tells a convincing and well-organised story about the data and topic |
|  | 10 | A good balance between analytic narrative and illustrative extracts is provided |
| Overall | 11 | Enough time has been allocated to complete all phases of the analysis adequately, without rushing a phase or giving it a once-over-lightly |
| Written report | 12 | The assumptions about, and specific approach to, thematic analysis are clearly explicated |
|  | 13 | There is a good fit between what you claim you do, and what you show you have done – i.e., described method and reported analysis are consistent |
|  | 14 | The language and concepts used in the report are consistent with the epistemological position of the analysis |
|  | 15 | The researcher is positioned as active in the research process; themes do not just ‘emerge’ |
